# Impact of genital Chlamydia trachomatis infection on reproductive outcomes among infertile women undergoing tubal flushing: a retrospective cohort at a fertility centre in Uganda

**DOI:** 10.1186/s40738-019-0069-5

**Published:** 2019-12-12

**Authors:** Anthony Kayiira, Daniel Zaake, Michael Webba Lwetabe, Peter Sekweyama

**Affiliations:** 1 Life Sure Fertility and Gynaecology centre, St. Francis Hospital Nsambya, postgraduate medical school, Uganda Martyr’s University Nkozi, Nsambya, Uganda; 2 St. Francis Hospital Nsambya, Nsambya, Uganda

**Keywords:** Tubal flushing, *C. Trachomatis*, Tubal-factor, Infertility, Low-income

## Abstract

**Background:**

The impact of current *C. trachomatis* on clinical pregnancy and live birth rates among women undergoing tubal flushing is largely unknown. This study aimed to investigate whether current female genital *C. trachomatis* infection affects the chance of achieving a clinical pregnancy and a live birth, among infertile women undergoing tubal flushing, at a fertility centre in Uganda.

**Methods:**

A retrospective Cohort study at a peri-urban fertility centre. A total of 253 eligible women with tubal factor infertility, who underwent tubal flushing, were enrolled and categorised according to their exposure to current genital *C. trachomatis* infection. These women were followed up for a period of 12 months, with the primary outcome measure being clinical pregnancy and live birth. Secondary outcome measures included pregnancy loss and procedural related adverse events.

**Results:**

Exposure to current genital *C. trachomatis* infection reduced chance of clinical pregnancy (adjusted relative risk 0.42; 95% confidence interval, 0.18–0.96) and a live birth (adjusted relative risk 0.37; 95% confidence interval, 0.14–0.95) after tubal flushing. Women with current *C. trachomatis* infection had an increased risk of adverse events (adjusted relative risk, 1.20; 95% confidence interval, 1.08–1.34). However, current *C. trachomatis* infection did not affect the risk of spontaneous abortion and ectopic pregnancy.

**Conclusion:**

Current genital *C. trachomatis* infection in women with tubal factor infertility, undergoing tubal flushing, lowers their chance of pregnancy and live birth.

## Background

Sub-Saharan Africa is among the regions with the highest burden of infertility, contributing to nearly a quarter of all infertile couples in the world [1]. A female factor is identified as the cause of infertility in 35% of couples [2] and fallopian tube pathology is the underlying cause of infertility in 30–35% of infertile women [3]. Moreover, majority of tubal pathology results from an antecedent *C. trachomatis* infection [4–8].

In low income settings, interventions such as tubal flushing present a low cost, accessible and minimally invasive alternative for diagnosis and treatment of tubal factor infertility. Although there is a lot of debate about the effectiveness of tubal flushing as therapy for sub fertility [9–13], a metanalysis by Mohiyiddeen, Hardiman [14] found that tubal flushing with oil soluble contrast media increased the chance of pregnancy and live birth compared to no intervention. Furthermore, there was no determination on the difference between the oil soluble and water soluble contrast regarding rates of ongoing pregnancy [14].

In Uganda, use of tubal flushing for the treatment of subfertility is prolific but safety and efficacy data is lacking. Occasional anecdotal reports on the results of tubal flushing are inconsistent and do not report on live birth rates. There is a need for criteria to select women who are most likely to benefit from tubal flushing while limiting the risk for complications.

Considering that 70–80% of *C. trachomatis* infections go unrecognised [15], persistent infection may increase the risk for pelvic inflammatory disease, tubal factor related infertility and ectopic pregnancy [15–17]. Moreover, all these sequelae can increase the severity of any pre-existing tubal pathology which may reduce the chance of pregnancy after tubal flushing. In addition, genital *C. trachomatis* infection has been associated with an increased risk of miscarriage [18]. However, there is a paucity of data on the impact of genital *C. trachomatis* on clinical pregnancy and live birth rates among women undergoing tubal flushing. There is uncertainty as to whether screening for current *C. trachomatis* infection, presents an opportunity to guide the judicious prescription of tubal flushing among women with tubal factor infertility in low-income settings. Therefore, this study aimed to investigate whether current female genital *C. trachomatis* infection affects the chance of achieving a clinical pregnancy and a live birth among infertile women undergoing tubal flushing at a fertility centre in Uganda.

## Methods

### Study design

Retrospective cohort study from 1stJanuary 2012 to 1stJanuary 2017. Data was collected from treatment charts at Lifesure fertility and gynaecology centre in Uganda.

### Study setting

Lifesure fertility and gynaecology centre is a private clinic located in peri urban Kampala. Patients are referred there for diagnostic evaluation and therapy in fertility and assisted reproduction. The centre is run by a team of three gynaecologists, specialised in reproductive medicine, and a team of dedicated fertility nurses and clinical embryologists who offer support. At the first consultation, all women and their partners go through an assessment which includes (i) detailed history (pertaining information on; age, menstrual health, parity, abortions, infertility duration, marital status, social economic status, surgery, sexually transmitted infections, fertility treatment and contraceptive use), (ii) physical examination, (iii) radiological evaluation (including; hysterosalpingography (HSG) and transvaginal ultrasound) and serum hormonal assays. For men, in addition to history taking and physical examination, a seminal analysis is done. Contributory causes of infertility are documented in the treatment chart before treatment is prescribed. When the aetiology of infertility is related to abnormalities in tubal patency as denoted on the HSG, tubal blockage is graded into grade (I) with moderate spillage of contrast, (II) with minimal spillage of contrast and (III) with no spillage of contrast. If a woman meets the criteria for tubal flushing i.e. she has tubal factor related infertility in absence a male factor, she will have a genital *C. trachomatis* antigen test. The centre uses a direct binding monoclonal based immunochromatographic assay (Cypress diagnostics, Belgium) for the visual detection of *C. trachomatis* antigen from endocervical samples [for procedural details see Additional file 1]. As standard, women with genital *C. trachomatis* infection, and their sexual partners, receive a single dose (1 g) of oral Azithromycin as treatment before the tubal flushing procedure. The tubal flushing procedure is carried out using aqueous media in a set of three timed series i.e. once a day for three consecutive days between day 6 and day 10 of the menstrual cycle [for procedural details see Additional file 2]. Following tubal flushing, conception is managed expectantly with a prescription; to have sexual intercourse three times a week. The couple is followed up at 6 months or until conception whichever comes first. If the couple has not conceived by 6 months, a repeat tubal flushing is at the discretion of the couple. If the woman is not pregnant, regardless of whether a second tubal flushing is performed, the follow up is extended for another 6 months. If the couple has not conceived at 12 months, they may opt for either tubal surgery or in vitro fertilization (IVF).

### Study population

All women aged 18 to 40 years, with tubal factor related infertility and undergoing tubal flushing from 1^st^ January 2012 to 1^st^ January 2017, were included in the cohort provided they had been screened for current genital *C. trachomatis* infection.

Data was collected on 446 women, of whom 253 were included in the cohort at baseline. [See Additional file 3: Figure S1]. The reasons for exclusion were comorbid ovulatory dysfunction (*n* = 73), submucosal or large (> 6 cm) intramural uterine fibroids (*n* = 10), mullerian duct anomalies (*n* = 4), abnormal semen analysis (*n* = 50), presence of hydrosalpinx (*n* = 10), and if treatment charts were missing information on outcomes (*n* = 46).

### Exposure to genital chlamydia

Data on genital *C. trachomatis* exposure was obtained from the treatment charts, where results of routine genital *C. trachomatis* antigen screening are documented.

### Outcomes

The patients were followed up at 6-month intervals to make a total period of 12 months from the first tubal flushing. The primary outcomes in this study were: [1] Clinical pregnancy, measured as a population rate and defined as an intrauterine pregnancy with a confirmed cardiac activity using ultrasound at 7–8 weeks of amenorrhea [2]. Live birth, measured as a population rate and defined as a live born at 28 weeks or more of gestation. The secondary outcomes were: [1] Pregnancy loss, measured as a population rate, and defined as any gestation not reaching 28 weeks. These included miscarriages and ectopic gestations [2]. Adverse events at tubal flushing, also measured as a population rate, and defined as documentation of either bleeding, pelvic pain or acute pelvic inflammation at and or within 7 days of tubal flushing.

Information about all the outcomes were abstracted from the treatment charts and all the primary outcomes were considered if they occurred within 12 months (52 weeks) after the first tubal flushing.

### Confounding variables

Directed acyclic graphs (DAGs) were used to identify variables that could potentially confound the primary outcomes, using the principles outlined by Howards [19]. Therefore we adjusted for the following confounders in our statistical models: age at the time of tubal flushing (years; 19–25, 26–30, 31–35, 36+), duration of infertility (years; < 2, 2–5, 6+), previous infertility treatment (binary; tubal flushing, ovulation induction, alternative medicine and assisted reproductive technologies), parity (categorical; multiparous, nulliparous), history of genital infection (binary) and highest attained education level (categorical; low which was primary or no education and high which was secondary, technical or vocational education). Although the type of tubal blockage, degree of tubal blockage and presence of uterine filling defects were associated with genital *C. trachomatis* exposure, they did not fulfil the criteria for confounding variables [19]. Further analysis found that they fulfilled the criterial for mediators [20] because they seemed to be on the casual path through which *C. trachomatis* exposure affected the primary outcomes.

### Statistical methods

Abstracted data was entered and analysed in STATA 15 (StataCorp).

#### Participant characteristics

Abstracted data on participant demographics and outcome modifiers was grouped into respective exposure (exposed to current genital *C. trachomatis*) and non-exposure groups. The characteristics were calculated into proportions and presented as percentages. A chi-square statistic was used to determine any differences between the exposed and non-exposed women.

#### Outcomes

A modified Poisson regression, using a generalised linear model with robust standard errors, was used to model the estimated risk of current genital *C. trachomatis* exposure on getting a clinical pregnancy and a live birth after tubal flushing. The models were adjusted for the previously mentioned confounding variables and the estimates presented as crude and adjusted relative risk with their 95% confidence intervals. Poisson regression models were run to estimate the risk of current genital *C. trachomatis* exposure on the secondary outcomes, pregnancy loss and adverse events.

#### Sub analysis

Mediation analysis was performed to determine the indirect effect of current genital *C. trachomatis* exposure on the risk of achieving a clinical pregnancy and live birth. The mediator variables were type of tubal blockage, degree of tubal blockage and presence of intrauterine filling defects. The product of coefficients method was used, and the resulting total indirect and direct effects were used to compute the proportion of the risk that was due to the either of the mediator variables. Bootstrapping was used to compute the standard errors and 95% confidence intervals to determine whether the mediation effects of the above-named variables were statistically significant.

### Ethical approval

This study was approved by the research and ethics committee, identification number UG-REC-020, and a waiver for informed consent was obtained before patient file abstraction. All personal identifiers were anonymised, and abstraction forms kept in a secure location. All electronic data was password protected and uploaded on a secure computer.

## Results

In the study period, 253 women with tubal factor infertility had tubal flushing procedures and were followed for 12 months. The prevalence of current genital *C. trachomatis* among this population was 18.2%. All data for the variables listed in Table 1 were complete. Baseline data on lifestyle outcome modifiers i.e. alcohol consumption, smoking and body mass index, were inconsistently documented and omitted from analysis.

**Table 1:** Characteristics of the study population according to current genital *Chlamydia trachomatis* exposure

Genital Chlamydia exposure			
Characteristics	Negative (*N* = 207)	Positive (*N* = 46)	*P* value
Age group at start of treatment (%)			0.941
19–25	12.08	10.87	
26–30	42.51	39.13	
31–35	27.05	28.26	
36 or more	18.36	21.74	
Parity (nulliparous; %)	64.73	56.52	0.296
History of abortion (%)	37.2	26.09	0.153
Duration of infertility (%)			0.831
< 2 years	20.77	17.39	
2–5 years	62.32	63.04	
6 or more years	16.91	19.57	
Marital Status (%)			0.541
Single	14.49	13.04	
Married	75.85	71.74	
Cohabiting	9.66	15.22	
Highest attained education level ^a^ (%)			0.643
Low	8.70	10.87	
High	91.30	89.13	
Occupation ^b^ (%)			0.147
Unemployed	4.83	0.00	
Unskilled worker	1.45	4.35	
Skilled worker	93.72	95.65	
History of infertility treatment ^c^ (%)	92.27	97.83	0.173
History of gynaecological surgery ^d^ (%)	41.55	32.61	0.263
History of obstetrical surgery ^e^ (%)	11.11	15.22	0.436
History of genital infection (%)	2.90	36.96	0.000
Recent use of hormonal contraception ^f^ (%)	2.42	6.52	0.150
Type of tubal blockage (%)			0.002
Unilateral	36.71	13.04	
Bilateral	63.29	86.96	
Grade of tubal blockage ^g^ (%)			0.043
I. Moderate spillage	27.05	13.04	
II. Minimal spillage	47.34	45.65	
III. No spillage	25.6	41.3	
Presence of uterine filling defect (%)	10.14	23.91	0.011
Number of tubal flushing series in 12 months			0.004
One	88.41	71.74	
Two	11.59	28.26	

^a^Low: primary or no education. High: secondary, technical or vocational education

^b^Unskilled labour: work with no special training or experience. Skilled labour: work with special training and experience

^c^Previous treatment with either tubal flushing, ovulation induction, alternative medicine or assisted reproductive technologies

^d^Either myomectomy, tubal surgery, uterine instrumentation or ovarian cystectomy

^e^Either caesarean section or other obstetric surgical procedures

^f^Either oral contraceptive, subdermal implant, injectable contraceptive or intrauterine device

^g^Tubal patency as denoted on the HSG, grade (I) with moderate spillage of contrast, (II) with minimal spillage of contrast and (III) with no spillage of contrast

Table 1 depicts characteristics of the study population at enrolment for tubal flushing according to genital *C. trachomatis* exposure status. There was no difference between the exposed and non-exposed women for most of the baseline variables. Women with genital *C. trachomatis* were more likely to have a prior genital infection, bilateral tubal blockage, grade III tubal blockage, uterine filling defects and a second tubal flushing procedure.

Overall, regardless of exposure, women undergoing tubal flushing had a clinical pregnancy rate of 28.85% and a live birth rate of 24.9%. All the pregnancies among the women with current genital *C. trachomatis* occurred within 6 months of follow up, whereas 94.12% of the non-exposed women got pregnant within 6 months of follow up. Table 2 and Table 3 depict the proportions of clinical pregnancy and live birth according to genital *C. trachomatis* exposure status.

**Table 2:** Associations between genital *Chlamydia trachomatis* exposure, among women undergoing tubal flushing, and Clinical pregnancy rate

Chlamydia exposure	Total (n)	Cases (%)	cRR (95% CI)	aRR (95% CI)
Negative	207	32.85	1.00 (Reference)	1.00 (Reference)
Positive	46	10.87	0.33 (0.14–0.78)*	0.42 (0.18–0.96)*

*p value < 0.05; cRR: crude relative risk; aRR: adjusted relative risk

**Table 3:** Associations between genital *Chlamydia trachomatis* exposure, among women undergoing tubal flushing, and live birth rate

Chlamydia exposure	Total (n)	Cases (%)	cRR (95% CI)	aRR (95% CI)
Negative	207	28.50	1.00 (Reference)	1.00 (Reference)
Positive	46	8.70	0.30 (0.12–0.80)*	0.37 (0.14–0.95)*

*p value < 0.05; cRR: crude relative risk; aRR: adjusted relative risk

Women with current genital *C. trachomatis* and undergoing tubal flushing, had a reduced chance of achieving a clinical pregnancy (adjusted relative risk [aRR] 0.42; 95% confidence interval [CI], 0.18–0.96) and a live birth (aRR 0.37; 95% CI, 0.14–0.95) compared to the non-exposed women (Table 2 and Table 3).

Among women undergoing tubal flushing, there was no association between current genital *C. trachomatis* infection and pregnancy loss (aRR 0.77; 95% CI, 0.10–5.68), as depicted in Table 4. Women with genital *C. trachomatis* were more likely to experience adverse events following tubal flushing (aRR 1.20; 95% CI, 1.08–1.34) compared to the non-exposed women (Table 5).

**Table 4:** Associations between genital *Chlamydia trachomatis* exposure, among women undergoing tubal flushing, and pregnancy loss

Chlamydia exposure	Total (n)	Cases (%)	cRR (95% CI)	aRR (95% CI)
Negative	207	4.35	1.00 (Reference)	1.00 (Reference)
Positive	46	2.17	0.50 (0.06–3.87)	0.77 (0.10–5.68)

**Table 5:** Associations between genital *Chlamydia trachomatis* exposure, among women undergoing tubal flushing, and procedure-related adverse events

Chlamydia exposure	Total (n)	Cases (%)	cRR (95% CI)	aRR (95% CI)
Negative	207	80.19	1.00 (Reference)	1.00 (Reference)
Positive	46	91.30	1.14 (1.02–1.27)*	1.20 (1.08–1.34)*

*p value < 0.05; cRR: crude relative risk; aRR: adjusted relative risk

In the sub analysis, the effect of current genital *C. trachomatis* infection on clinical pregnancy and live birth rate was partly indirect through the bilateral tubal blockage, grade (II and III) of tubal blockage and uterine filling defects. In a model with genital *C. trachomatis* infection and either of the mediator variables, grade (II and III) of tubal blockage had the highest mediation effect on clinical pregnancy (57.3%) and live birth (57%). Uterine filling defects had the lowest mediation effect on clinical pregnancy (11.7%) and live birth (12.6%). The mediation coefficients for all the mediator variables, obtained by bootstrapping, were statistically significant as presented in Table 6.

**Table 6:** Results from mediation analysis

Mediator variable	Indirect effect (%) ^a^	Mediator coefficient	Bias	Bootstrap standard error	Bias corrected 95% Confidence interval
Bilateral tubal blockage	51.3 ^b^; 46.7 ^c^	−0.113 ^b^; −0.092 ^c^	0.007 ^b^ 0.005 ^c^	0.026 ^b^; 0.231 ^c^	[− 0.149, − 0.055] ^b^ [− 0.125, − 0.049] ^c^
Grade II and III tubal blockage	57.3 ^b^; 57 ^c^	−0.2 ^b^; − 0.178 ^c^	0.008 ^b^ 0.005 ^c^	0.086 ^b^; 0.079 ^c^	[−0.368, − 0.057]^b^ [− 0.319, − 0.039] ^c^
Uterine filling defects	11.7 ^b^; 12.6 ^c^	−0.026 ^b^; − 0.025 ^c^	−0.003 ^b^ − 0.002 ^c^	0.018 ^b^; 0.015 ^c^	[−0.069, − 0.003] ^b^ [− 0.064, − 0.004] ^c^

^a^effect of genital *Chlamydia trachomatis* infection on outcome that is indirect via the mediator variable.; ^b^mediation on clinical pregnancy; ^c^mediation on live birth

## Discussion

This cohort study of 253 Ugandan women with tubal factor infertility, undergoing tubal flushing, showed that women with current genital *C. trachomatis* infection were less likely to achieve a clinical pregnancy and a live birth. Women with genital *C. trachomatis* infection were more likely to suffer from procedure-related adverse events although there was no association with pregnancy loss.

An 18.2% prevalence of genital *C. trachomatis* in this population is nearly 5 -fold that previously reported in Uganda [21] and the current global prevalence estimate [22]. Notably, global prevalence estimates exclude high risk populations of which infertile women belong. Nonetheless, similar prevalence estimates have been reported among low risk African populations i.e. 17.8% [23], 16.5% [24], 16.1% [25]. However, higher prevalence estimates have been reported among women in low resource settings with tubal factor infertility i.e. (35.3%) [8] and 75% [26]. Both studies [8, 26] utilised systemic antibody against *C. trachomatis* proteins which are more sensitive for previous *C. trachomatis* exposure unlike direct local antigen tests which detect current infection.

Overall, a clinical pregnancy rate of 28.85% and a live birth rate of 24.9% following tubal flushing with aqueous media among women with tubal factor infertility was similar to that reported by Spring, Barkan [27]. However, Spring, Barkan [27] conducted diagnostic tubal flushing among a heterogenous population of women with infertility. Interestingly, among participants with grade III tubal blockage (no contrast spillage on HSG) there were 5(6.94%) clinical pregnancies and 4(5.56%) live births after tubal flushing (not shown). Therefore, tubal flushing is still a viable low-cost intervention among women with tubal factor infertility.

Although several studies have linked *C. trachomatis* infection to infertility [5, 8, 26, 28], there are no reports on the impact of active genital *C. trachomatis* infection on outcomes among women undergoing tubal flushing. This study found an association between active genital *C. trachomatis* infection and reduced clinical pregnancy and live birth rate among women who underwent tubal flushing. Among subgroup of participants with grade III tubal blockage (not shown), there was only 1(5.26%) clinical pregnancy and live birth in those with genital *C. trachomatis*. This was lower than 4(7.55%) clinical pregnancies and 3(5.66%) live births in the non-exposed group.

The reduction in the chance of clinical pregnancy and live birth could be explained by the fact that women with genital *C. trachomatis* infection were more likely to have bilateral tubal blockage and severe degrees of tubal blockage, both of which may adversely affect pregnancy outcomes, at baseline. Indeed, mediation analysis found these factors along the casual path between genital *C. trachomatis* and adverse pregnancy outcomes. Even though exposed women received treatment for *C. trachomatis* before tubal flushing, the pervasive and persistent nature of the *C. trachomatis* rendered them susceptible to chronic upper genital tract inflammation and autoimmune phenomena [29]. The other studies, performed among women undergoing IVF and utilising Chlamydial serological testing, found conflicting results i.e. reduced pregnancy rate [30, 31] and no difference in pregnancy rate [32].

The lack of association between genital *C. trachomatis* exposure and pregnancy loss (abortions or ectopic pregnancy), as seen by the wide confidence intervals, arises from the fact that the sample size did not achieve statistical power to detect these differences. However, several observational studies have reported a positive association between genital *C. trachomatis* and spontaneous abortions [18, 33, 34] or ectopic pregnancy [15–17].

The fact that women with genital *C. trachomatis* were likely to have bilateral and severe tubal disease may explain their increased risk of procedure-related adverse events i.e. pelvic pain and bleeding. However, acute pelvic inflammatory disease which is a known complication of ascending *C. trachomatis* during instrumentation [35], was not reported. This could have been mitigated by the treatment for *C. trachomatis* received prior to the procedure.

The results from this study imply that for couples with tubal factor infertility and active genital *C. trachomatis*, tubal flushing is less likely to result in a live birth. This chance is reduced even with appropriate antibiotic treatment. An escalation of fertility treatment to IVF, in these couples, may be justified in order to offer a better chance at a live birth.

The strength of this study lies in the fact that it is the first in a low income setting where tubal flushing for treatment of subfertility is prolific and not guided by proper evidence. Information concerning most of the confounding variables was present in the treatment charts and thus allowed us carry out sensitive statistical models for the outcomes. Nonetheless, smoking, alcohol use and body mass index data was inconsistently reported. Excluding women with ovulatory dysfunction and male factor related infertility allowed us to purely assess the impact of *C. trachomatis* infection among women undergoing tubal flushing. This study was limited by a retrospective design which carries the risk of misclassification and missing information. Over 36.96% of the participants with current genital *C. trachomatis* had a history of genital infection compared to 2.9% of participants in the non-exposed group. A sub analysis (not shown) found an association between past genital infection and clinical pregnancy and live birth. Although we adjusted for this confounder in a robust poisson regression model, the marked skewed distribution of this variable may create residual confounding. A small sample size limited the study’s ability to make inferences on secondary outcomes. Direct *C. trachomatis* antigen tests have limited sensitivity for upper genital tract infections and are inferior to nuclear amplification tests [36]. Nonetheless, the direct binding monoclonal based immunochromatographic assay utilised by the clinic has a reported sensitivity of 75 to 85% and specificity of 98 to 99% (reported by Cypress diagnostics, Belgium). The frequency of sexual intercourse under expectant management following tubal flushing was prescribed but not assessed and thus could have confounded the results. This study only reports on current *C. trachomatis* infection. Missing information on subclinical or previous genital *C. trachomatis* infections could have confounded the results.

## Conclusion

Results from this study suggest that current *C. trachomatis* infection in women with tubal factor infertility, undergoing tubal flushing, lowers their chance of pregnancy and live birth. This reduction occurs irrespective of pre-procedure antibiotic treatment. Therefore, women with tubal factor infertility should be screened for current *C. trachomatis* infection before undergoing tubal flushing. This will enable clinicians to advise couples and prescribe a low-cost intervention judiciously among women who are most likely to benefit with minimal risk. Larger cohorts in low-income settings are needed to confirm these findings and make inferences on the association with spontaneous abortions and ectopic pregnancies among women undergoing tubal flushing.

## Supplementary information

**Additional file 1.** The genital Chlamydia trachomatis antigen test.

**Additional file 2.** The tubal flushing procedure.

**Additional file 3: Figure S1.** A flow chart for the study population. Ag: Antigen.

## Data Availability

All data generated or analysed during this study is available from the authors upon reasonable request.

## Abbreviations

DAG: Directed acyclic graphs
HSG: Hysterosalpingography
IVF: In vitro fertilisation
