# Supplementary material for: Impact of genital Chlamydia trachomatis infection on reproductive outcomes among infertile women undergoing tubal flushing: a retrospective cohort at a fertility centre in Uganda
Source: Fertil Res Pract. 2019 Dec 12;5:16. doi: 10.1186/s40738-019-0069-5 (PMC6909488; doi:10.1186/s40738-019-0069-5)
Supplement: Supplementary file 1 — Additional file 1. The genital Chlamydia trachomatis antigen test. [file 40738_2019_69_MOESM1_ESM.docx]

**The genital Chlamydia trachomatis antigen test**

The centre uses a direct binding monoclonal based immunochromatographic assay (Cypress diagnostics, Belgium) for the visual detection of *Chlamydia trachomatis* antigen from endocervical samples. It has a reported sensitivity of 75% to 85% and specificity of 98% to 99% (reported by Cypress diagnostics, Belgium) . In the procedure, the endocervical sample is obtained by aseptic technique with the woman in lithotomy position and with the aid of a sterile Cusco’s speculum to visualise the cervix. A sterile microbial sample swab is introduced into the endocervical canal to a depth of 2cm and rotated once for 360 degrees. The sample is then placed into an extraction tube containing extraction solution A. After two minutes, extraction solution B is added to the tube and 3 drops (approximately 150 µl) of extracted sample is added to the sample well. The membrane is pre-coated with anti-genus specific lipopolysaccharide (LPS) monoclonal antibody on the test band (T) region and goat anti-mouse antibody on the control band (C) region. During testing, the sample is allowed to react with the colloidal gold particles which have been coated with monoclonal anti-Chlamydia antibody and then it migrates laterally across the membrane by capillary action. If the sample contains Chlamydia antigen, a coloured band with a specific antibody- Chlamydia antibody-colloidal gold particle complex will form on the membrane in the test band (T) region. If Chlamydia antigen is not present, only a pink line forms on control band (C) region. To serve as a procedural control, a coloured band at the control band (C) region will always appear regardless of the presence of Chlamydia antigen.
