# Supplementary material for: Impact of genital Chlamydia trachomatis infection on reproductive outcomes among infertile women undergoing tubal flushing: a retrospective cohort at a fertility centre in Uganda
Source: Fertil Res Pract. 2019 Dec 12;5:16. doi: 10.1186/s40738-019-0069-5 (PMC6909488; doi:10.1186/s40738-019-0069-5)
Supplement: Supplementary file 2 — Additional file 2. The tubal flushing procedure. [file 40738_2019_69_MOESM2_ESM.docx]

**The tubal flushing procedure**

The tubal flushing procedure is carried out in three timed series between day 6 and day 10 of the menstrual cycle. The procedure is done under aseptic technique with the woman in low dorsal lithotomy position. Baseline vitals are taken, and analgesia is provided with intramuscular analgesia using a single dose of 100mg Tramadol. A sterile Cusco’s speculum is inserted into the vagina to expose the cervix and the uterus is sounded with a curved Sim’s uterine sound to ascertain the depth and orientation of the uterine cavity. A straight metallic cervical cannula is inserted into the cervical canal, to no more than 2cm depth. When the cannula is snug tight in the cervix, aqueous based sterile media (4 ml of Gentamicin sulphate at 40mg/ml, 2ml of reconstituted Hydrocortisone at 50mg/ml and 14ml of sterile water for injection) to the limit of 20 ml is slowly infused into the cannula at a rate of 1ml per minute. The level of resistance to flow, back flow, bleeding and intensity of pain is noted and documented. Post procedure vitals are taken, and the woman can rest until she feels ready to ambulate home. All women receive antibiotic prophylaxis after tubal flushing. This consists of oral Ampiclox 500mg three times a day for 5 days and oral Metronidazole 400mg twice a day for 5 days.
