# Supplementary material for: Impact of genital Chlamydia trachomatis infection on reproductive outcomes among infertile women undergoing tubal flushing: a retrospective cohort at a fertility centre in Uganda
Source: Fertil Res Pract. 2019 Dec 12;5:16. doi: 10.1186/s40738-019-0069-5 (PMC6909488; doi:10.1186/s40738-019-0069-5)
Supplement: Supplementary file 3 — Additional file 3: Figure S1. A flow chart for the study population. Ag: Antigen. [file 40738_2019_69_MOESM3_ESM.docx]

Figure S1: A flow chart for the study population. Ag: Antigen

**Potential study population**

N = 446 women

**Eligible study population at baseline**

N = 253 women

Chlamydia Ag test

**Excluded**

- Ovulatory dysfunction (n = 73)
- Submucosal or large (> 6cm) intramural uterine fibroids (n = 10)
- Mullerian duct anomalies (n = 4)
- Abnormal semen analysis (n = 50)
- Presence of hydrosalpinx (n=10)
- Treatment charts missing information on outcomes (n = 46)

Positive

(Exposed group)

N = 46 women

Negative

(Non-exposed group)

N = 207 women

Tubal flushing

Follow up at 6 months

Analysis

N = 46 women

Analysis

N = 207 women

Follow up at 12 months
